# Supplementary material for: Activation of M1 cholinergic receptors in mouse somatosensory cortex enhances information processing and detection behaviour
Source: Commun Biol. 2024 Jan 2;7:3. doi: 10.1038/s42003-023-05699-w (PMC10761830; doi:10.1038/s42003-023-05699-w)
Supplement: Supplementary file 2 — Supplementary Information [file 42003_2023_5699_MOESM2_ESM.pdf]

Supplementary Information

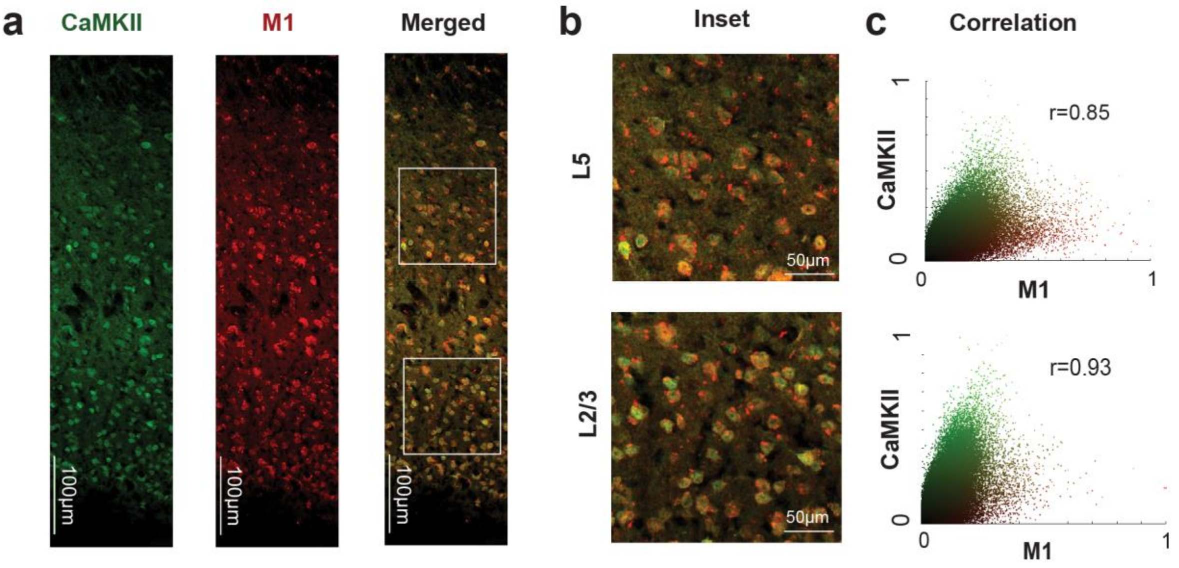

Supplementary Fig. 1: M1 expression on vS1 excitatory neurons. **a)** Co-localisation of M1 (red) with CaMKII (green, a marker for excitatory neurons) across layers of the vS1 (Scale bar = 100µm). **b)** Inset - Co-localisation of M1 and CaMKII in Layer 5 and Layer 2/3 of the vS1 cortex (Scale bar = 50µm). **c)** Pearson's correlation coefficients depicting the co-localisation of M1 and CaMKII in Layer 5 and Layer 2/3.

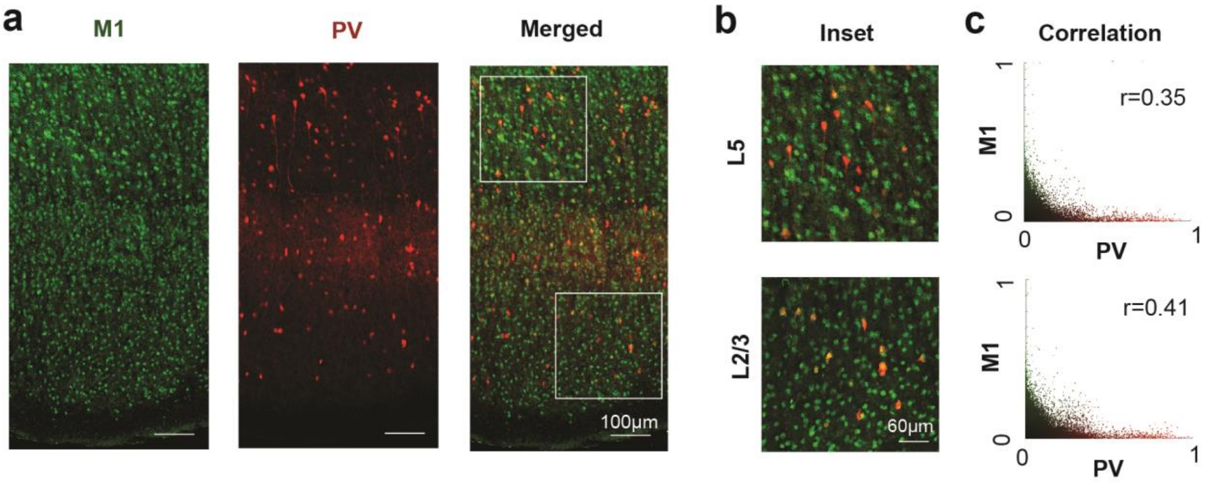

Supplementary Fig. 2: M1 expression of on the vS1 inhibitory neurons. **a)** Co-localisation of M1 (green) with PV (red, a marker for a subtype of inhibitory interneurons) across layers of the vS1 (Scale bar = 100µm). **b)** Inset - Co-localisation of M1 and PV in Layer 5 and Layer 2/3 of the vS1 (Scale bar = 60µm). c) Pearson’s correlation coefficients depicting the co-localisation of M1 and PV in Layer 5 and Layer 2/3.

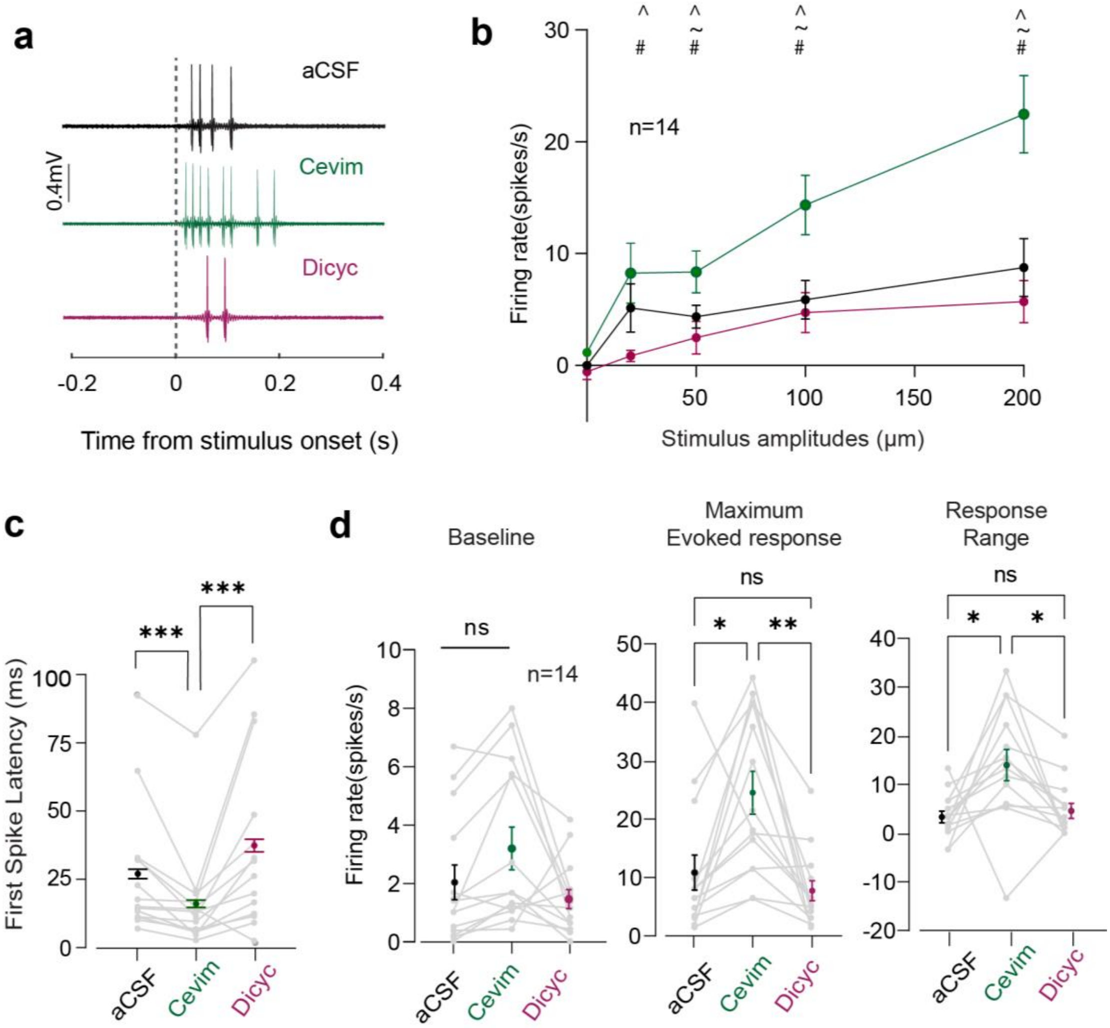

Supplementary Fig. 3: M1 activation enhances evoked responses in vS1 neurons. **a)** Raw voltage traces showing the spiking activity for an example neuron in response to a 200 μm whisker vibration after application of aCSF (black, control), Cevimeline hydrochloride (green, M1 agonist) or Dicyclomine hydrochloride (magenta, M1 antagonist). The grey vertical dotted line represents the stimulus onset at time zero. **b)** The input/output function for all neurons under aCSF, Cevimeline and Dicyclomine conditions. Each dot represents the mean firing rate across all neurons (n=14, 5 mice, Friedman test with Dunn's multiple comparision), ~-Significant statistical difference between Cevimeline and control; #- between Cevimeline and Dicyclomine; ^- between Dicyclomine and control. **c)** First spike latencies calculated in a 100-ms window post stimulus presentation (200 μm, n=14, Friedman test with Dunn's multiple comparison). **d)** M1-induced changes in baseline firing, evoked response and the response range. Grey dots indicate single neurons and the solid dots represent the means. The vertical lines represent the SEM. ***$p<0.001$, **$p<0.01$, *$p<0.05$.

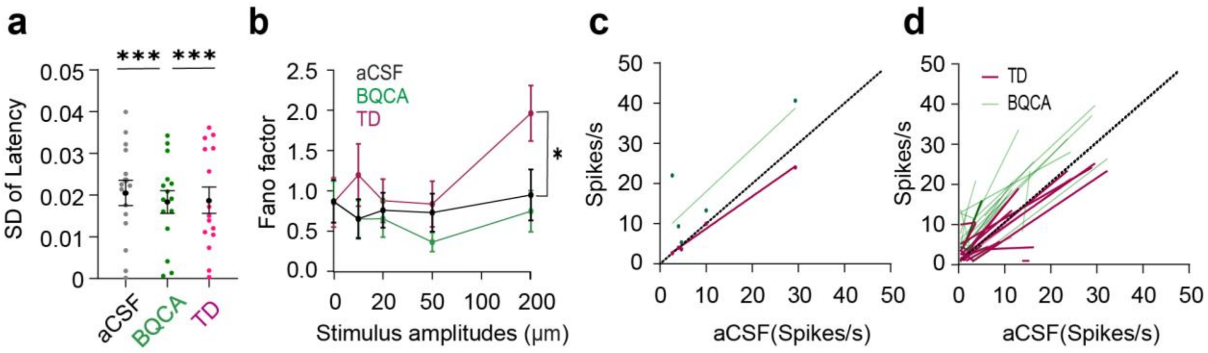

Supplementary Fig. 4: **a)** Standard Deviation of the first spike latencies in Fig. 1f show reduced variability after M1 activation with BQCA (green) and increased variability after M1 inhibition with TD ($p<0.001$, Wilcoxon signed-rank test). **b)** Fano Factors of the spike latencies for all stimulus amplitudes shown for aCSF (black), BQCA (Green) and TD (Magenta). There is a significant increase in the Fano Factors at the highest stimulus amplitude (200μm) after applying TD, which indicates that blocking M1 increases the variability in response latencies. Introducing BQCA reduced the Fano factors (*$p<0.05$, Friedman test with Dunn's multiple comparision). **c)** The neuronal response of one example neuron after M1 activation (BQCA, green) and inhibition (TD, magenta) is plotted against that response at the aCSF condition. The green and the magenta lines illustrate the line of best fit used to calculate the slopes and intercepts of the neuronal response; the black dotted line is the line of equivalence. **d)** The best-fitted lines plotted for all neurons M1 activation (BQCA, green) and inhibition (TD, magenta); the black dotted line is the line of equivalence. The vertical lines represent the SEM.

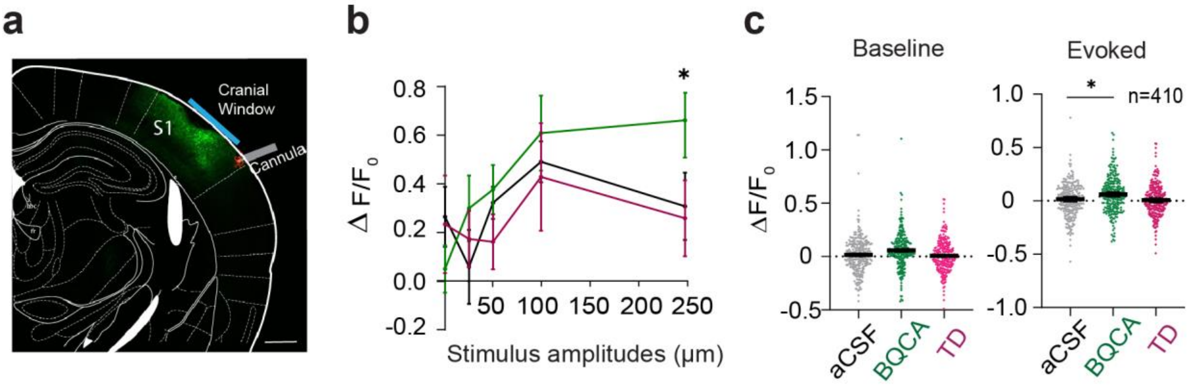

Supplementary Fig. 5: **a)** A histology image showing the position of the implanted cannula stained with DiI (red) along with GCaMP7f expression (Green) in the vS1 (Scale bar = 500µm). **b)** Characterisation of M1 modulation on neuronal population using 2-photon Calcium imaging in anaesthetised animals. Changes in $\Delta F/F_0$ measured in a 1 s window after stimulus onset in the aCSF (black), BQCA (green) and TD (magenta) conditions for all stimulus amplitudes. The dots represent the mean $\Delta F/F_0$; the error bars represent the SEM (n=410, 4 mice). **c)** M1 modulation of the baseline (left) and maximum evoked (right) response. Every dot represents a neuron. Black lines and error bars indicate the mean and SEM across neurons (n = 410, 4 mice, *$p<0.01$, Friedman Test with Dunn's multiple comparison). The vertical lines represent the SEM.

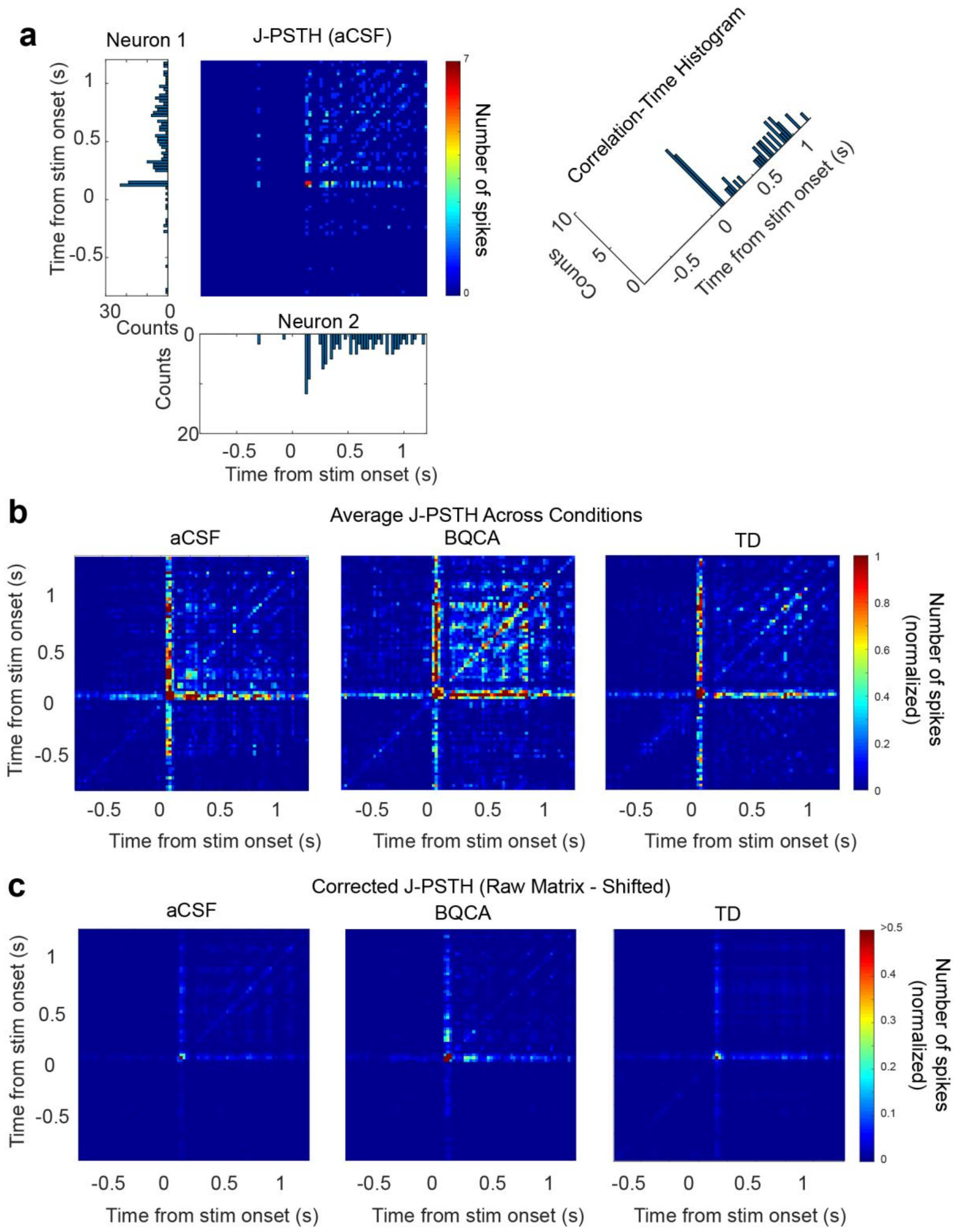

Supplementary Fig. 6: Joint peristimulus time-histogram (J-PSTH) analysis. **a)** For every trial, spikes from one neuron are arranged on the x- axis, and spikes from another neuron are arranged on the y-axis. Coincident spikes over the course of the trial are recorded in the J-PSTH matrix. This is repeated for every trial and presented as the heatmap. The 45-degree diagonal in this matrix is illustrated as a correlation time histogram on the right. **b)** The cumulative J-PSTH for all neuronal pairs in the control condition (aCSF, left), after M1 activation (BQCA, middle) and M1 inhibition (TD, right). The J-PSTH is normalized by the product of standard deviations of PSTHs [1]. **c)** To correct for coincident spikes related to stimulus presentation, a shift predictor matrix is subtracted from the raw J-PSTH, and the entire quantity is normalized by the product of standard deviations of PSTHs.

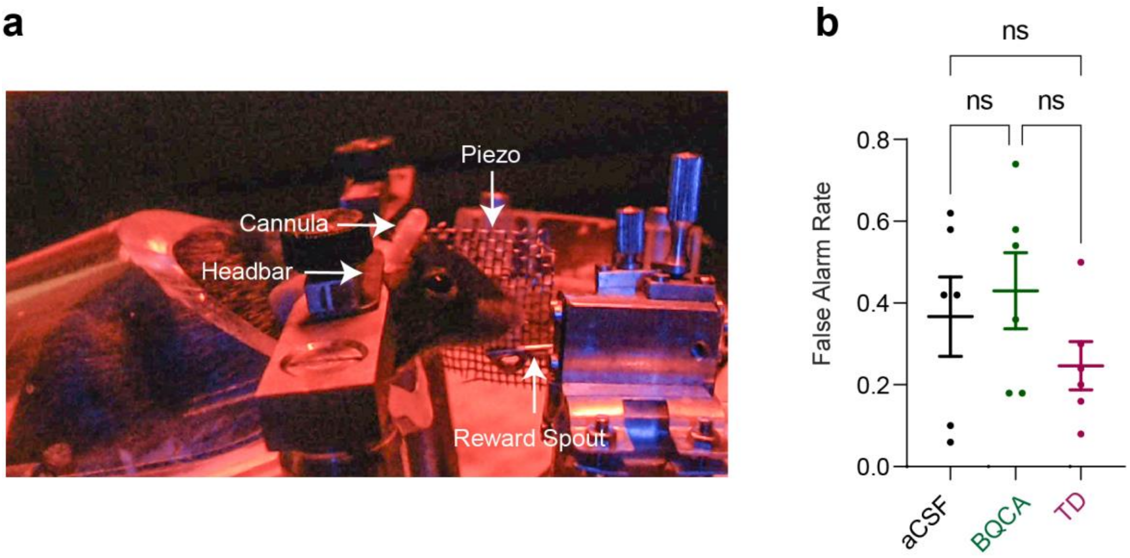

Supplementary Fig. 7: **a)** The awake detection task set-up: An example head-fixed mouse engaged in the detection task. A cannula is implanted over the vS1 for drug perfusion during the task. A wire mesh connected to a piezoelectric stimulator is used to stimulate the whiskers at different amplitudes and a reward spout delivers a drop of sucrose in hit trials. **b)** The average false alarm rates across 6 mice show no significant changes with M1 modulations (n = 6, p>0.05, Friedman test with Dunn's multiple comparison). The vertical lines represent the SEM.

Supplementary References:

1. Aertsen, A. M. H. J., Gerstein, G. L., Habib, M. K. & Palm, G. Dynamics of neuronal firing correlation: modulation of 'effective connectivity'. *J. Neurophysiol.* **61**, (1989).
