# Supplementary material for: Activation of M1 cholinergic receptors in mouse somatosensory cortex enhances information processing and detection behaviour
Source: Commun Biol. 2024 Jan 2;7:3. doi: 10.1038/s42003-023-05699-w (PMC10761830; doi:10.1038/s42003-023-05699-w)
Supplement: Supplementary file 3 — Description of Additional Supplementary Files [file 42003_2023_5699_MOESM3_ESM.docx]

**Description of Additional Supplementary Files**

**File name:** Supplementary Movie 1

**Description:** Cross-correlations across neurons over time in an anaesthetised mouse.

**File name:** Supplementary Movie 2

**Description:** M1 mediated modulation of cross-correlations across neurons over time (awake).
