# Supplementary material for: Activation of M1 cholinergic receptors in mouse somatosensory cortex enhances information processing and detection behaviour
Source: Commun Biol. 2024 Jan 2;7:3. doi: 10.1038/s42003-023-05699-w (PMC10761830; doi:10.1038/s42003-023-05699-w)
Supplement: Supplementary file 6 — Reporting Summary [file 42003_2023_5699_MOESM6_ESM.pdf]

nature portfolio

Corresponding author(s): Ehsan Arabzadeh

Last updated by author(s): Dec 5, 2023

Reporting Summary

Nature Portfolio wishes to improve the reproducibility of the work that we publish. This form provides structure for consistency and transparency in reporting. For further information on Nature Portfolio policies, see our Editorial Policies and the Editorial Policy Checklist.

Statistics

For all statistical analyses, confirm that the following items are present in the figure legend, table legend, main text, or Methods section.

n/a	Confirmed	
☐	☒	The exact sample size (*n*) for each experimental group/condition, given as a discrete number and unit of measurement
☐	☒	A statement on whether measurements were taken from distinct samples or whether the same sample was measured repeatedly
☐	☒	The statistical test(s) used AND whether they are one- or two-sided<br>*Only common tests should be described solely by name; describe more complex techniques in the Methods section.*
☒	☐	A description of all covariates tested
☐	☒	A description of any assumptions or corrections, such as tests of normality and adjustment for multiple comparisons
☐	☒	A full description of the statistical parameters including central tendency (e.g. means) or other basic estimates (e.g. regression coefficient) AND variation (e.g. standard deviation) or associated estimates of uncertainty (e.g. confidence intervals)
☐	☒	For null hypothesis testing, the test statistic (e.g. *F*, *t*, *r*) with confidence intervals, effect sizes, degrees of freedom and *P* value noted<br>*Give P values as exact values whenever suitable.*
☒	☐	For Bayesian analysis, information on the choice of priors and Markov chain Monte Carlo settings
☒	☐	For hierarchical and complex designs, identification of the appropriate level for tests and full reporting of outcomes
☒	☐	Estimates of effect sizes (e.g. Cohen's *d*, Pearson's *r*), indicating how they were calculated

Our web collection on statistics for biologists contains articles on many of the points above.

Software and code

Policy information about availability of computer code

Data collection	PsychToolbox for MATLAB (R2019 and R2020) was used for stimulus presentation. The two-photon data were collected using Thorlabs software<br>(ThorImageLS V.3).
Data analysis	GraphPad Prism V9.5.1 (https://www.graphpad.com/scientific-software/prism/) was used for statistical analysis of juxtacellular electrophysiology data. For two-photon Calcium imaging data, Suite2p (https://github.com/MouseLand/suite2p) and ImageJ (https://imagej.nih.gov/ij/) was used to process the raw data. Analysis was done using custom MATLAB scripts (R2019 and R2020).

For manuscripts utilizing custom algorithms or software that are central to the research but not yet described in published literature, software must be made available to editors and reviewers. We strongly encourage code deposition in a community repository (e.g. GitHub). See the Nature Portfolio guidelines for submitting code & software for further information.

Data

Policy information about availability of data

All manuscripts must include a data availability statement. This statement should provide the following information, where applicable:

- Accession codes, unique identifiers, or web links for publicly available datasets
- A description of any restrictions on data availability
- For clinical datasets or third party data, please ensure that the statement adheres to our policy

The data generated in this study is available at: https://osf.io/rd8b4/. The code used in this study has been published in an open-access format at: https://github.com/MishraWricha/Cholinergic-M1-receptors-in-sensory-procesing

Research involving human participants, their data, or biological material

Policy information about studies with human participants or human data. See also policy information about sex, gender (identity/presentation), and sexual orientation and race, ethnicity and racism.

Reporting on sex and gender	NA
Reporting on race, ethnicity, or other socially relevant groupings	NA
Population characteristics	NA
Recruitment	NA
Ethics oversight	NA

Note that full information on the approval of the study protocol must also be provided in the manuscript.

Field-specific reporting

Please select the one below that is the best fit for your research. If you are not sure, read the appropriate sections before making your selection.

☒ Life sciences ☐ Behavioural & social sciences ☐ Ecological, evolutionary & environmental sciences

For a reference copy of the document with all sections, see nature.com/documents/nr-reporting-summary-flat.pdf

Life sciences study design

All studies must disclose on these points even when the disclosure is negative.

Sample size	For juxtacellular electrophysiology experiments, we included 23 neurons from 17 animals. For two-photon experiments, more than 900 neurons were included from 6 mice. Power analysis was not conducted to set the sample size. Instead, the sample size was based on previous work from the lab and the literature which suggests 900 neurons would provide sufficient power to find moderate to large effects on neuronal response using two-photon imaging. For behavioral experiment, 6 mice were trained in the detection task.
Data exclusions	Two-photon calcium imaging sessions with large motion artifacts were excluded from analysis.
Replication	For 2-photon calcium imaging experiments, we performed 5 recording sessions for every mouse in each drug condition (aCSF, BQCA or TD). We also replicated these findings in over 400 neurons from 4 anesthetized animals leading to a total of 40 recording sessions for each drug condition. For behavioural experiments, all 6 animals had 5 recording sessions with each drug condition.
Randomization	The drug conditions were pseudo-randomly presented during the experiments.
Blinding	The study used a within-subject design with the same neurons examined across conditions. The experimenters were aware of the drug conditions used in the study.

Reporting for specific materials, systems and methods

We require information from authors about some types of materials, experimental systems and methods used in many studies. Here, indicate whether each material, system or method listed is relevant to your study. If you are not sure if a list item applies to your research, read the appropriate section before selecting a response.

Materials & experimental systems

n/a	Involved in the study	
☐	☒	Antibodies
☒	☐	Eukaryotic cell lines
☒	☐	Palaeontology and archaeology
☐	☒	Animals and other organisms
☒	☐	Clinical data
☒	☐	Dual use research of concern
☒	☐	Plants

Methods

n/a	Involved in the study	
☒	☐	ChIP-seq
☒	☐	Flow cytometry
☒	☐	MRI-based neuroimaging

Antibodies

Antibodies used	Primary: Goat anti-M1 AChR (Abcam Cat#ab77098), Rabbit anti-PV (Abcam Cat# ab11427), Anti-CaMKII (Abcam, Cat#ab32678). Secondary: Donkey Anti-goat 568 (Abcam Cat#ab175704), Donkey Anti-goat 488 (Abcam Cat#ab150129), Donkey Anti-rabbit 568 (Abcam Cat#ab175475) and Goat anti-rabbit 488 (Cat#ab150077).
Validation	Goat anti-M1 AChR is suitable for western blots and immunofluorescence, Rabbit anti-PV is suitable for immunofluorescence, Anti-CaMKII is suitable for western blots and immunofluorescence .

Animals and other research organisms

Policy information about studies involving animals; ARRIVE guidelines recommended for reporting animal research, and Sex and Gender in Research

Laboratory animals	Male and female C57BL6J mice were used in the study. The mice were bred in the Australian Phenomics Facility which were originally obtained from Jackson Laboratories. Mice were kept in cages attached to Tecniplast Smart Flow. The system keeps the cage temperature at 22 °C. The humidity was typically around 40-50%. For two-photon experiments, mice were transfected at 4-5 weeks with recordings starting 3-4 weeks later, and lasted for 2 to 3 weeks. For behavioural experiments, 4-6 week old mice were trained in the detection task for 1 weeks followed by 3 weeks of recording with drug applications. For juxtacellular experiments, 4-12 week old mice were used. Mice were ethically culled at a maximum of 12 weeks of age.
Wild animals	NA
Reporting on sex	The study included both males and female animals.
Field-collected samples	NA
Ethics oversight	All methods were performed in accordance with the protocol approved by the Animal Experimentation and Ethics Committee of the Australian National University (AEEC 2019/20 and 2022/16).

Note that full information on the approval of the study protocol must also be provided in the manuscript.
